# Defect in Brnym1, a magnesium-dechelatase protein, causes a stay-green phenotype in an EMS-mutagenized Chinese cabbage (*Brassica campestris* L. ssp. *pekinensis*) line

**DOI:** 10.1038/s41438-019-0223-6

**Published:** 2020-01-01

**Authors:** Nan Wang, Yun Zhang, Shengnan Huang, Zhiyong Liu, Chengyu Li, Hui Feng

**Affiliations:** Department of Horticulture, Shenyang Agricultural University, Shenyang, China

**Keywords:** Plant breeding, Plant breeding, Plant breeding, Plant breeding

## Abstract

Leaf color is an important target trait in Chinese cabbage breeding programs. Leaf yellowing may reduce crop commercial and nutritional values. Some plants with the “stay-green” trait maintain leaf greenness during senescence and even after death. Stay-green Chinese cabbage may be a focal point of future breeding projects because it could improve crop quality and yield and prolong shelf life. A new stay-green mutant, *non-yellowing mutant 1* (*nym1*), was identified in Chinese cabbage derived from an ethyl methane sulfonate (EMS)-mutagenized population. The mutant had stay-green characteristics and a higher chlorophyll content than the wild-type during leaf senescence. The stay-green trait in the mutant Chinese cabbage was controlled by the recessive gene *Brnym1*. MutMap and KASP analyses showed that *Brnym1* (*BraA03g050600.3C*) encodes an mg-dechelatase (SGR protein), which might be the causal gene of the mutation in Chinese cabbage. A nonsynonymous single nucleotide base substitution (G to A) in the third exon of *Brnym1* caused an amino acid substitution from L to F in the highly conserved domain of the magnesium-dechelatase. Ectopic overexpression showed that the *BrNYM1* gene of wild-type Chinese cabbage complemented the SGR-defective stay-green mutant *nye1-1* of *Arabidopsis*. The magnesium-dechelatase activity in the *nym1* mutant was significantly downregulated compared to that in the wild type. *Brnym1* was relatively upregulated in the mutant during late senescence, and BrNYM1 was localized to the chloroplasts. These results indicate that *Brnym1* (*BraA03g050600.3C*) is the causal gene of the stay-green mutation and could be of particular significance in the genetic improvement of Chinese cabbage.

## Introduction

The stay-green trait generally refers to plants that retain their leaf greenness during senescence and even after death^1^. Stay-green mutants were classified into five types by Thomas and Howarth^2^. In type A plants, the onset of senescence is delayed, but its progression is normal. In type B plants, the timing of the onset of senescence is normal, but its progression is delayed. In type C plants, the plants are defective in terms of chlorophyll degradation. These plants are called cosmetic stay-greens. In type D plants, the plant leaf dies in response to sudden freezing or drying, but its chlorophyll does not degrade rapidly. Thus, the plant appears green (pseudostay-green). In type E plants, the plant has a stay-green phenotype as it accumulates a large amount of chlorophyll, which requires a relatively long time to degrade. Plant types A and B have delayed senescence, prolonged photosynthetic activities, and possibly higher yields than wild-type plants. They are known as “functional stay-greens”^3^ and are associated with drought or heat tolerance^4^ and disease resistance^5^. The functional stay-green trait may be used to breed high-yield and stress-resistant crop varieties for agricultural production. Type C “stay-green” plants preserve their leaf color by retaining chlorophyll. However, neither their photosynthesis nor their senescence is affected. Thus, they are called “nonfunctional (or cosmetic) stay-greens”^3^. Cosmetic stay-green mutants have been used to investigate the molecular mechanisms of stay-green genes in various crops.

Chinese cabbage (*Brassica rapa* ssp. *pekinensis*) originated in central China. It is an important vegetable crop in Asia and is used in fresh and processed food products. Its leaf color usually changes from green to yellow during senescence. At that time, its nutrient content and, by extension, its commercial value decrease and may result in severe postharvest loss. The functional stay-green trait is an important trait to improve postharvest storage and prolong the shelf life of Chinese cabbage. The cosmetic stay-green trait may enhance the sensory quality, commodity value, and shelf life of Chinese cabbage. Cosmetic stay-greens may be useful for the genetic improvement of leafy vegetables in breeding programs.

The identification of stay-green mutants may also elucidate the underlying molecular mechanisms of the stay-green trait and facilitate genetic crop improvement. The first known stay-green mutant was *Pisum sativum* (pea) with green cotyledons. It was used by the botanist Gregor Mendel to establish the laws of genetics. Various stay-green cultivars have been identified in natural populations and by mutagenetic screening and breeding programs^5^. The stay-green varieties FS854 of *Zea mays*^2^ and SNU-SG1 of *Oryza sativa*^6^ were identified in breeding programs. A similar phenotype was described for a natural soybean *(Glycine max*) population^7^. A *non-yellowing1* (*nye1-1*) *Arabidopsis thaliana* mutant was induced by the fast-neutron process^8^. Several horticultural stay-green mutants have also been reported. Kerr identified the tomato stay-green mutant *green flesh* (*gf*) in 1956^9^. The impaired yellowing of a *Phaseolus vulgaris* (snap bean) stay-green mutant during leaf senescence has been investigated^10^. The chlorophyll retainer mutant *cl* was reported in pepper, and the mutation within it inhibits chlorophyll degradation during fruit ripening^11^. A pigment analysis suggested that ripening-related chlorophyll degradation was impaired in a *Citrus* stay-green mutant^12^. The natural mutant *Nye* causing stay-green leaves was recently identified in pakchoi^13^.

Mapping and cloning the stay-green genes are preconditions to the study of its molecular mechanism and applications. The impairment of genes encoding chlorophyll catabolic enzymes (CCEs) may result in the stay-green phenotype. Cloning *NYC1*, encoding Chl *b* reductase, and its homolog, *NOL*, in rice confirmed this mechanism^14,15^. *HCAR* encodes 7-hydroxymethyl Chl *a* reductase and was detected in the *Arabidopsis hcar* stay-green mutant. In *hcar*, 7^1^-OH-Chl *a* is not converted to Chl *a*^16^. The *PPH* genes encoding pheophytinase were cloned from *pph* stay-green rice and *Arabidopsis* mutants^17,18^. A mapping analysis of *PAO* uncovered genetic defects in rice *lls1* and *Arabidopsis acd1* stay-green mutants^19,20^. The genetic defect in certain stay-green mutants has been attributed to mutations in *STAY-GREEN* (*SGR*), including Mendel’s green cotyledon mutant^21^ (2-aa insertion in *PsSGR*), tomato *gf*^22^ (point mutations), and pepper *cl*^23^ (point mutations in *CaSGR*). Analyses of rice stay-green mutants^24^ and the *Arabidopsis nye1-1* mutant^8^ indicated that defects in the *SGR* orthologs were responsible for the stay-green mutant phenotypes.

Historically, defects in *SGR* have been attributed to abnormal PAO activity or expression since several SGR-deficient mutants exhibited low PAO activity, but other catabolic enzymes were unaffected^25,26^. Later, reports showed no difference in *PAO* expression between SGR-deficient mutants (rice *sgr-2*^8^, *Arabidopsis nye1-1*^21^) and wild type. This discrepancy was explained by the fact that their SGR protein extraction efficiencies differed^27,28^. SGR proteins have been reported to be highly conserved among various plant species and may be located in the chloroplast^29^. SGRs could be regulatory proteins controlling chlorophyll degradation during senescence. Sakuraba et al.^30^ proposed that SGRs recruit CCEs based on their ability to bind light-harvesting complex II (LHCII) and their interactions with CCEs. Shimoda et al. (2016) showed that SGRs encode mg-dechelatases^31^. STAY-GREEN is a functional mg-dechelatase that extracts magnesium from free chlorophyll and chlorophyll complexes and therefore participates in chlorophyll and photosystem degradation. The magnesium-dechelatase catalyzes the first step of Chl *a* degradation^32^. The *C*-terminal domain of NYE1/SGR contains a cysteine-rich motif (CRM) essential for SGR function in Chl degradation, mg dechelation, and conformational changes in SGR via disulfide bond formation^33^.

Here, we identified a stay-green Chinese cabbage in an EMS-mutagenized population. It retained leaf greenness longer and had higher chlorophyll content than the wild type during senescence and even after death. We discovered by MutMap and KASP that the mutation responsible for delayed degreening might be a nonsynonymous base pair change in *Brnym1* (mg-dechelatase). Compared to wild type, the mutant had significantly downregulated magnesium-dechelatase activity. *BrNYM1*, the *Brnym1* allele in wild type, fully rescued the stay-green phenotype in the SGR-defective *nye1-1* mutant of *Arabidopsis*. These results revealed that the function of *BrNYM1* was to regulate chlorophyll breakdown*. Brnym1* was induced by senescence. To the best of our knowledge, this study is the first to clone the stay-green gene in Chinese cabbage. The findings herein may help develop new tools for the genetic improvement of horticultural crops.

## Results

### Phenotypic characterization and genetic analysis of nym1

**Fig. 1:**
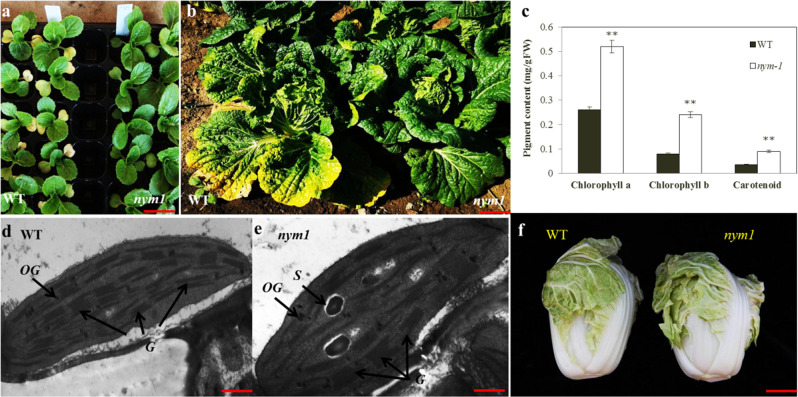
Morphological characterizations of wild-type (“FT”) and *nym1* mutant plants. **a** Phenotypic characterizations of wild-type and mutant cotyledons. Plants 20 DAS (days after sowing). Bars = 3 cm. **b** Phenotypic characterizations of wild-type and *nym1* rosette leaves. Plants were at the field-grown heading stage (50 DAS). Bars = 10 cm. **c** Pigment content of wild type and mutant bottom leaves. Plants 60 DAS. Ten plants from the wild type and “*nym1*” lines with the same growth potential were randomly selected. The second leaves from the bottom were sampled. **Significantly different at *P* = 0.01 by the *t* test. **d**, **e** Transmission electron microscopy (TEM) of WT and mutant bottom leaves. The plant stage was the same as that for Fig. 1c. Three leaf specimens were sampled per plant. Fifteen sections were viewed by TEM. Twelve cells were examined and photographed per section. S starch granule, OG and arrow osmiophilic plastoglobuli, G granum. Bars = 5 μm. **f** Phenotypic characterizations of wild type and mutant leafy heads. Plants 70 DAS. Left panel: wild type; right panel: *nym1*. Bars = 7 cm.

The *nym1* mutant was identified among M_2_ lines in an EMS-mutagenized population of the double haploid “FT” line. Compared to “FT” (wild type), the mutant had leaves that did not turn yellow during senescence or after death at the cotyledon and rosette stages, respectively (Fig. 1a, b). There were no other phenotypic differences between the mutant and wild-type “FT”. The leafy head of the mutant was the same size as that of the wild type (Fig. 1f). The mutant exhibited a stable, inheritable stay-green phenotype throughout its entire growth period (Figs. 1a, b), and its stems and siliques also remained green at harvest (Fig. S1).

To detect the genetic lesions associated with Chl degradation, the leaf pigments were measured and compared between *nym1* and “FT”. The chlorophyll content was relatively low in the senescent “FT” leaves, but approximately half of the pigment persisted in the senescent *nym1* leaves. The carotenoid content in “FT” was less than that in the *nym1* mutant (Fig. 1c). There were no apparent differences between the mutant and wild type in terms of their bottom leaf chloroplast morphology at 40 d after sowing (Fig. 1d, e). The numbers of chloroplasts and stroma lamellae were similar for both *nym1* and “FT” (Fig. 1d, e).

Phenotypic segregation analysis showed that all F_1_ progeny had yellow leaves. For the “FT” × *nym1* F_2_ plants, 576 and 186 had yellow and stay-green leaves, respectively. This observation was consistent with the predicted 3:1 segregation (*χ*^2^ = 0.14). Seventy-three F_1_ × “FT” individuals had yellow leaves. In contrast, 57 yellow and 52 stay-green plants were produced by the F_1_ × *nym1* backcross. This 1:1 segregation ratio was consistent with expectations (*χ*^2^ = 0.23). A single recessive nuclear gene, *Brnym1*, is responsible for the stay-green mutation in *nym1* (Table S1).

### Identification of the Brnym1 causal gene by MutMap

**Fig. 2:**
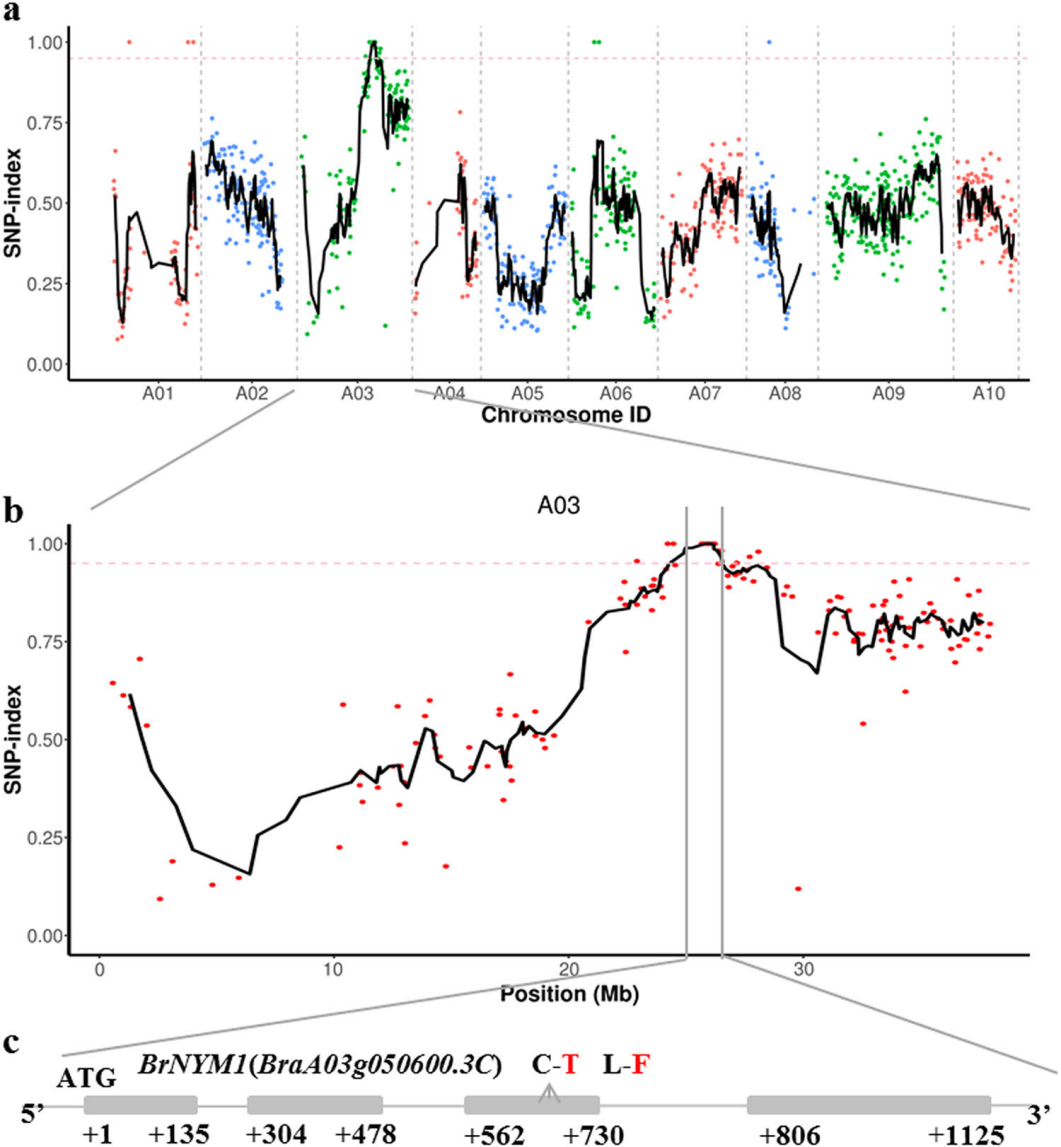
SNP index plot from MutMap and gene structure of *BrNYM1*. **a** SNP index of ten chromosomes plotted by MutMap sliding window analysis. The *x*-axis represents the positions of ten chromosomes. The *y*-axis represents the SNP index. The dotted pink line is the index (0.95) threshold line. Black lines were created by averaging SNP indices from a moving window spanning five consecutive SNPs and moving window one SNP at a time. **b** SNP index plot from MutMap identifying the candidate region on chromosome A03. Black and dotted pink lines are described above. **c** Candidate genes in the mapping region. Gray boxes and arrows represent genes and mutation sites, respectively. Sequence analysis revealed one SNP (C in wild-type; T in mutant) in the third exon resulting in an amino acid mutation (L in wild type; F in mutant).

A modified MutMap method was used to identify the mutant gene. After resequencing, we obtained 32,254 Mb (98.58% coverage) and 14,167 Mb (98.28% coverage) clean reads for one wild-type plant and the mutant pool, respectively. For the latter, 98.69% of the reads were aligned to the Chinese cabbage v. 3.0 reference genome. A total of 98,086 single-nucleotide polymorphisms (SNPs) were detected between the wild-type and mutant pools. To validate correlation accuracy, we restricted the SNPs as follows: (a) those supporting <5 or >120 reads were filtered from WT; (b) those supporting <5 or >240 reads were filtered from the mutant pool; (c) those that were not typical EMS-mutagenized type mutations (C→T or G→A) were filtered out; and (d) only those for homozygous wild-type and mutants with different typing were conserved. We obtained 1379 SNPs for the index analysis. For all ten Chinese cabbage chromosomes, the SNP index was calculated in a one-SNP increment and a five-SNP window (Fig. 2a). We localized a 1.59-Mb candidate region (24,908,848–26,503,461) on chromosome A03 when SNP index = 0.95 was selected as the threshold (Fig. 2b). Ten SNP mutations occurred, but only 25,986,068 and 26,145,830 were localized to the exon, causing the nonsynonymous base pair change (Fig. 2c; Table S2).

### Identification of causal SNPs

For the recessive trait, mutations in the exon causing missense or nonsense mutations more readily resulted in an extreme phenotype than mutations in the intron or intergenic region. To confirm the causal SNP for the stay-green phenotype, the mutated SNPs in the exon were used in KASP to design a specific primer for genotyping analysis of the 190 F_2_ individuals. The genotypic assay revealed that SNP 25,986,068 of *BraA03g050600.3C* cosegregated with the phenotype in the F_2_ segregation population. The stay-green plants had a T:T genotype (Fig. S2a), while the plants with a yellowing phenotype had a C:T or C:C genotype. In contrast, recombinants were found at SNP 26,145,830. Both the T:T and C:T genotypes were detected in the stay-green plants (Fig. S2b). Thus, SNP 26,145,830 of *BraA03g050790.3C* did not cosegregate with the stay-green phenotype (Table S3). The gene annotation in the *Brassica* database indicated that *BraA03g050600.3C* encodes magnesium-dechelatase (AtNYE1/STAY-GREEN1), which regulates chlorophyll degradation in *A. thaliana* leaf senescence^8^. The physical location of *BraA03g050600.3C* was on the peak of the linkage interval for this stay-green trait (Fig. 2b). Therefore, we presumed that *BraA03g050600.3C* was the candidate *Brnym1* gene.

### Function verification of BrNYM1

**Fig. 3:**
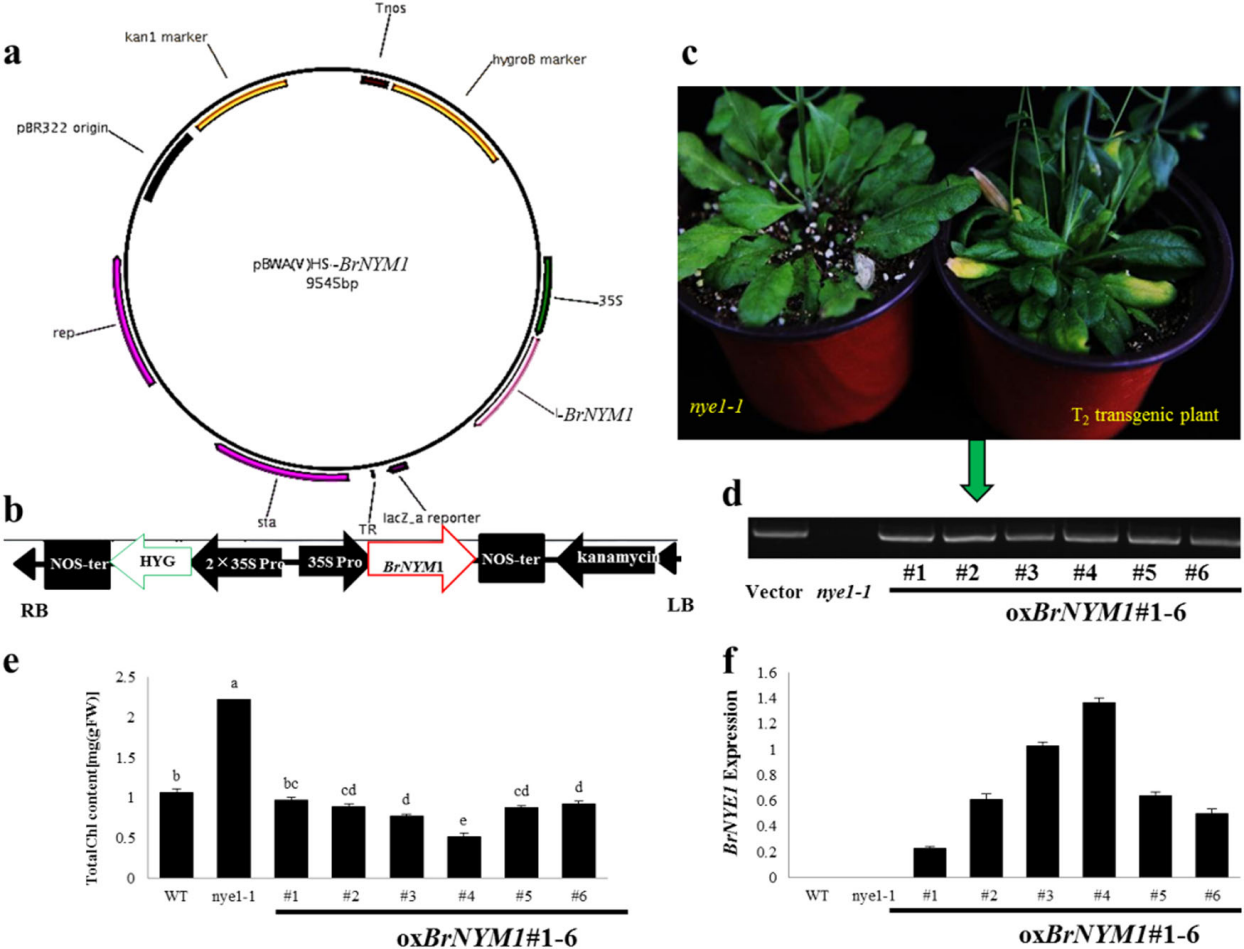
Transformation and phenotype of transgenic *Arabidopsis thaliana* plants transformed with *35SBrNYM1*. **a** Binary vector map of pBWA(V)BS–*BrNYM1*. **b** Diagram of components in the binary vector pBWA(V)BS–*BrNYE1*. RB right border, LB left border, NOS-pro nopaline synthase promoter, NOS-ter nopaline synthase terminator, HYG hygromycin, 35S-pro CaMV 35S promoter. **c** Morphology of representative T_2_ transgenic *A. thaliana* plants and stay-green mutant *nye1-1*. Left: *nye1-*1 with stay-green leaves; right: T_2_ transgenic plants with yellow phenotype. Plants 40 DAS. **d** PCR-based DNA genotyping of transgenic plants (1–6) with primers *35S_P* and *BrNYM1*. **e** Quantitative comparison of the total chlorophyll content in senescent leaves of Col-0, *nye1-1*, and transgenic plants (1–6) DPS v. 7.05 was used for variance analysis. Data were analyzed by Duncan’s multiple range test (DMRT). Different lowercase letters indicate significant differences (*n* = 3; *P* < 0.05). **f** Analysis of *BrNYM1* expression in Col-0, *nye1-1*, and transgenic plants (1–6).

To verify whether *BraA03g050600.3C* rescued the stay-green *nye1-1* (stay-green mutant) phenotype, a full-length, 795-bp CDS fragment of *BraA03g050600.3C* in “FT” was amplified and constructed into pBWA(V)BS-ccdB (Figs. 3a, b). The plasmid was introduced into *Agrobacterium tumefaciens* strain GV3101. *Nye1-1* was transformed by the standard dipping method. Thirty-six hygromycin-resistant plants were screened out and further confirmed from a specific gene fragment of *BraA03g050600.3C* (Fig. 3d). As with the Col-0 wild type, their leaves presented a yellowing phenotype during senescence (Fig. 3c right). On the other hand, *nye1-1* had a stay-green phenotype (Fig. 3c left). *BrNYM1* was significantly upregulated in ox*BrNYM1*#1–ox*BrNYM1*#6 transgenic plants (Fig. 3f). Moreover, the total chlorophyll content in their senescent leaves decreased to the wild-type level (Fig. 3e). Complementation confirmed the role of *BrNYM1* in regulating chlorophyll degradation. A defect in *BrNYM1* caused the stay-green phenotype, as reported for the SGRs of other species.

### Structural and phylogenetic analyses of BrNYM1

**Fig. 4:**
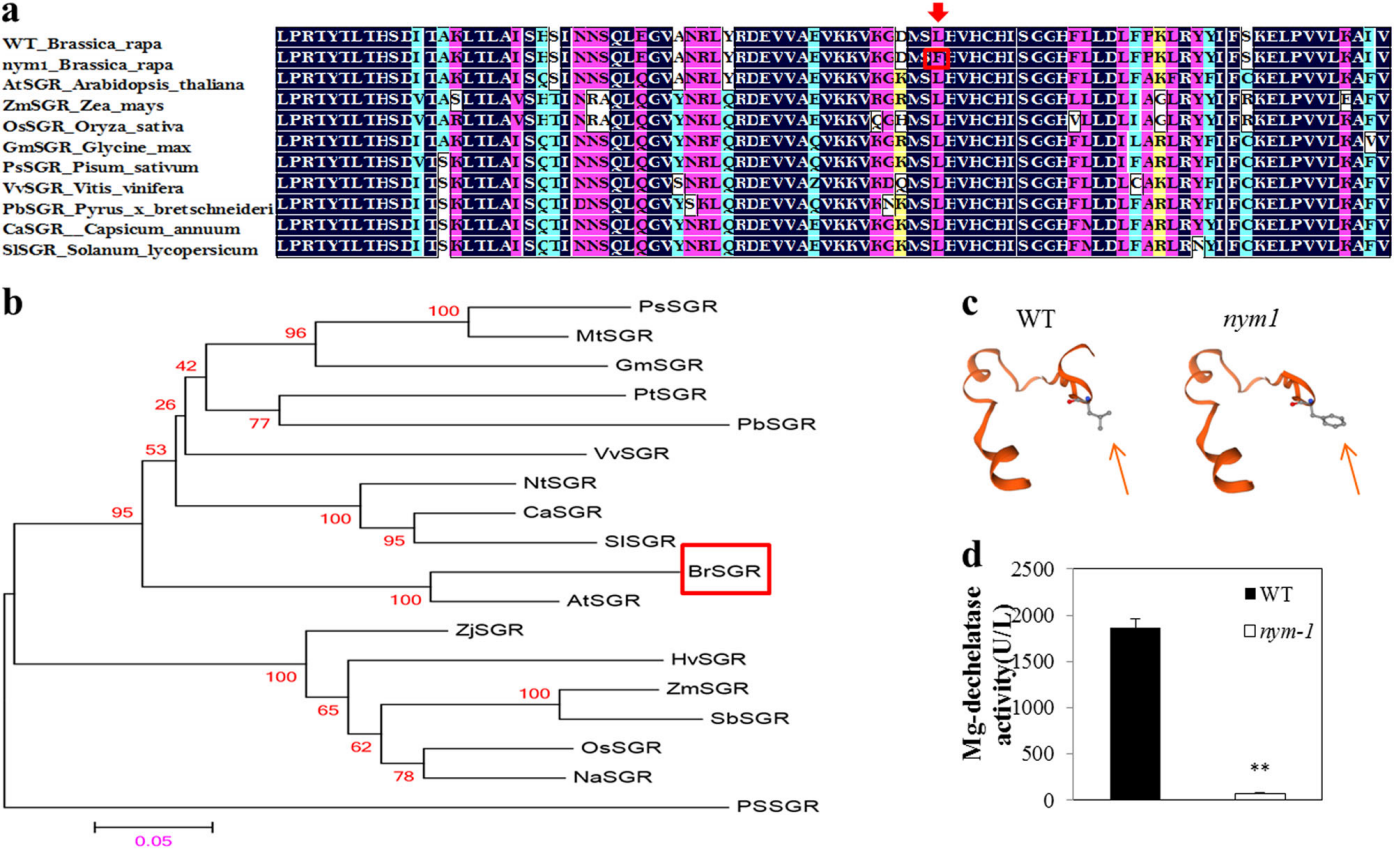
Partial sequence alignment and phylogenetic tree of BrNYM1. **a** SGR sequence alignment. Residues with 100% similarity are shaded in black. Those with ≥75% similarity are shaded in pink. Those with ≥50% similarity are shaded in blue. Those with ≥33% similarity are shaded in yellow. The SGR protein accession numbers are as follows: *Pisum sativum*, PsSGR (CAP04954); *Medicago truncatula*, MtSGR (AEE00201); *Glycine max*, GmSGR (AAW82959); *Populus trichocarpa*, PtSGR (EEE79433); *Pyrus×bretschneideri*, PbSGR (AEO19901); *Vitis vinifera*, VvSGR (AM455317); *Nicotiana tabacum*, NtSGR (ABY19382); *Capsicum annuum*, CaSGR (ACB56586); *Solanum lycopersicon*, SlSGR (ACB56587); *Arabidopsis thaliana*, AtSGR (AAW82962), *Zoysia japonica*, ZjSGR (AAW82961), *Hordeum vulgare*, HvSGR (AAW82955); *Zea mays*, ZmSGR (AAW82956); *Sorghum bicolor*, SbSGR (AAW82958); *Oryza sativa*, OsSGR (AAW82954); *Neosinocalamus affinis*, NaSGR (ADK56113); and *Picea sitchensis*, PSSGR (ABK22344). **b** Phylogenetic analysis of SGRs. **c** Partial 3D structure of BrNYM1 in wild type (left) and mutant (right). Amino acid sequences include SNPs. The red arrow represents different amino acid conformations at the mutant site. **d** Mg-dechelatase activity in “FT” and *nym1* during leaf senescence. The plant stage and sampling method were the same as those in Fig. 1c. Data were analyzed by a two-tailed *t* test. Significant differences were marked with an asterisk (*P* < 0.01). U/L = activity unit.

BrNYM1 has 265 amino acids, a molecular mass of 29.72 kDa, and a theoretical pI of 8.47. ChloroP analysis revealed that it has a chloroplast transit peptide region. The *nym1* mutation site in BrNYM1 was localized to a typical stay-green domain that is highly conserved among various plant species (Fig. 4a). Compared to wild type, the mutant has an extra benzene ring in the amino acid conformation of its mutation site (Fig. 4c). To establish whether this discrepancy affects protein function, an enzyme-linked immunosorbent assay (ELISA) was run. The magnesium-dechelatase activity was significantly lower in *nym1* than “FT” during senescence (Fig. 4d). An amino acid substitution may be responsible for the functional BrNYM1 defect in *nym1*. We constructed a phylogenetic tree to elucidate the evolutionary relationships among the BrNYM1 and SGR orthologs of various species. BrNYM1 was clustered with various dicots and closely related to AtNYE1/SGR (Fig. 4b).

### Spatiotemporal BrNYM1 transcription

**Fig. 5:**
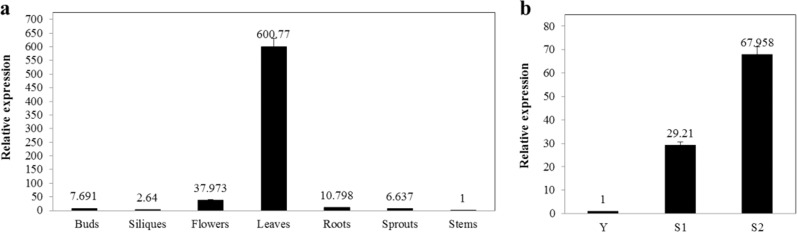
*BrNYM1* expression patterns. **a** Spatial *BrNYM1* expression in “FT” (wild type). **b** *BrNYM1* transcript levels at various developmental stages of “FT” (wild type). Y: young stage (sixth-leaf stage; 28 DAS); third true leaves of WT at the young stage were used to determine *BrNYM1* transcript levels; S1: onset of natural senescence stage (45 DAS); second true leaves from bottom were sampled. S2: progressive natural senescence stage (70 DAS); sampling method was the same as that for S1.

The relative *BrNYM1* transcription levels in various plant organs and developmental stages were measured by quantitative reverse transcription polymerase chain reaction (qRT-PCR) to determine the physiological function of this gene. For the wild type, the highest *BrNYM1* expression levels were found in the leaves, followed by the flowers, roots, buds, and sprouts. The lowest *BrNYM1* expression levels were established for the stems and young rosette leaves (Fig. 5a). Thus, BrNYM1 plays vital roles in Chinese cabbage leaves. In wild type, *BrNYM1* was upregulated during late senescence (Fig. 5b).

### BrNYM1 is localized to the chloroplasts

**Fig. 6:**
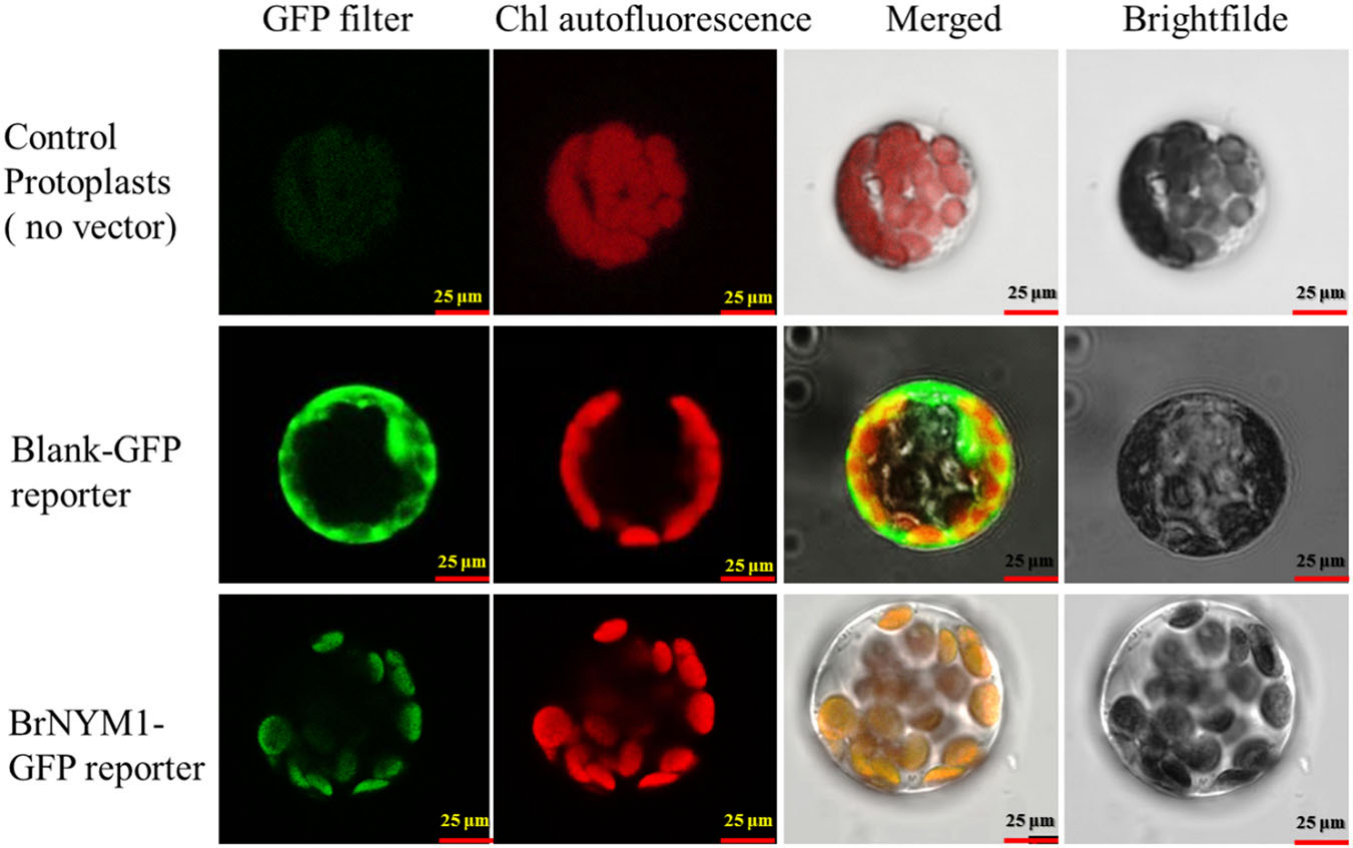
Localization of BrNYM1. Micrographs of *A. thaliana* protoplasts (top); blank-GFP reporter (middle); BrNYM1-GFP reporter (bottom). First column: GFP fluorescence. Second column: Chl autofluorescence. Third column: merged pictures of GFP and Chl autofluorescence. Fourth column: brightfield image. Three views were selected for electron microscope observation. For each section, >10 protoplasts were observed. Bars = 25 μm.

To detect whether BrNYM1 was located in the chloroplast, we constructed a fusion protein by joining its *C*-terminus to green fluorescent protein (GFP) and introduced the product into *A. thaliana* protoplasts. Green fluorescence signals were detected by monitoring transient GFP expression. GFP fluorescence was strong in the chloroplasts (Fig. 6, bottom) but weak in the blank control protoplasts (Fig. 6, top). In contrast, the GFP signal for the control was present in both the nuclei and cytoplasm (Fig. 6, middle). Thus, BrNYM1 localized mainly to the chloroplast.

## Discussion

Leaf color is important in Chinese cabbage because this crop is marketed as a green leaf product. The stay-green trait is a desirable agronomic trait in Chinese cabbage breeding. It delays leaf yellowing and improves the sensory quality and commodity value. Here, an EMS-induced stay-green mutant *nym1* was identified and used to map the stay-green gene. *Brnym1* (*BraA03g050600.3C*) encodes an AtNYE1/SGR1 protein responsible for the mutant stay-green trait. To the best of our knowledge, the present study is the first to identify a stay-green Chinese cabbage and clone the gene causing this trait. These findings may lay the foundation for unraveling the molecular mechanism underlying this phenotype.

The mutation inducing the stay-green characteristic could be a nonsynonymous base pair change in *Brnym1* (*SGR* encoding an mg-dechelatase). The previous sequence comparison showed a high degree of homology among the SGR proteins of various plant species. According to NCBI Blast analysis, SGR proteins have conserved domains in the stay-green superfamily. In their absence, a stay-green phenotype results^5^. Our sequence comparison revealed that the *nym1* mutation site in BrNYM1/BrSGR1 was localized to a stay-green domain and was highly conserved among different plant species (Fig. 4a). A nonsynonymous SNP in the third exon of *Brnym1* caused an amino acid substitution from Leu to Phe. The amino acid conformation in the mutant had an extra benzene ring at the mutation site located near the *C*-terminal domain of NYE1/SGR1 compared to that in wild type. The latter is essential for SGR activity in Chl degradation. The extra benzene ring in the mutant might obstruct disulfide bond formation and suppress mg dechelation^33^. In fact, we observed that magnesium-dechelatase activity was significantly downregulated in the mutant. Similarly, a single-base change from G to A was detected in the second exon of *OsSGR* in a rice *sgr* mutant. The mutation caused a Val→Met substitution and a defect in SGR function^29^. The SNP in *CaSGR* caused missense mutations in pepper *cl* mutants^22,23^. A complementation test revealed that a 6-bp insertion in the *PsSGRJI4* coding sequence (CDS) was responsible for the SGR function defect in JI2775^21^. The *BrNYM1* allele in WT fully rescued the stay-green phenotype in the SGR-defective *nye1-1* mutant of *Arabidopsis*. A complementation test in *Zoysia japonica* produced similar results. *ZjSGR* overexpression rescued the stay-green phenotype in *nye1-1*^34^. The present and previous studies demonstrated that *BrNYM1* affects the rate of tissue yellowing. Thus, a defect in it would be expected to cause the stay-green phenotype, as reported for the SGRs in other species. SGR function may depend on certain conserved amino acid residues. SNPs could substantially alter its 3D structure and, by extension, its activity.

EMS can induce hundreds to thousands of heritable single-base point mutations in one plant line. TILLING screens showed that the mutation densities ranged from 1:170–1:500 kb for diploid species^35^. Many other accompanying SNPs were also present in EMS-induced mutants. KASP is a flexible, cost-effective tool to distinguish alleles between parents and progeny in segregating populations. KASP has been used to verify causal genes in cloned regions of cucumber (*Cucumis sativus* L.)^31,36^. Our genotyping analysis showed that SNP 25,986,068 of *BraA03g050600.3C* cosegregated with the phenotype of the F_2_ segregation population. The stay-green plants had a T:T genotype (Fig. S2a), whereas the plants with the yellowing phenotype had a C:T or C:C genotype. In contrast, a recombinant was found at SNP 26,145,830, and the T:T and C:T genotypes were detected in the stay-green plants (Fig. S2b). Therefore, SNP 26,145,830 of *BraA03g050790.3C* did not cosegregate with the stay-green phenotype (Table S3).

In our study, the mechanism underlying *Brnym1* (*SGR*) was not elucidated. The amino acid sequence identity between Chinese cabbage SGR and its *Arabidopsis* homolog AtNYE1 was 84% (Fig. S3). It has been shown that AtNYE1 regulates chlorophyll degradation during leaf senescence in *Arabidopsis*^8^. Yeast two-hybrid (Y_2_H) analyses, *in vitro* and *in vivo* pulldown assays, and bimolecular fluorescence complementation (BiFC) in *Arabidopsis* revealed that SGR and five CCEs implicated in chlorophyll degradation were localized to LHCII and mutually interacted with each other. Dynamic SGR-CCE-LHCII protein interactions occurred on the thylakoid membrane^30^. Shimoda et al. (2016) used recombinant proteins from a wheat germ protein expression system to show that SGR/NYE encoded mg-dechelatase^32^. Matsuda et al. (2016) showed that the SGR/NYE function in unicellular algae is conserved in higher plants^37^. The *C*-terminal domain of NYE1^212–242^ has a CRM and is vital to SGR function in Chl degradation^33^. However, several unresolved issues merit investigation: (a) the mechanism by which stay-green plants avoid damage from pigment accumulation if certain chlorophyll metabolic intermediates are photosensitizers; (b) the nutritional quality of stay-green Chinese cabbage compared with that of the wild type; (c) whether the stay-green mutation also conserves mineral nutrients and photosynthate; (d) whether endogenous phytohormones affect the stay-green mutant; and (e) postharvest longevity in the mutant compared with that in the wild type. In future research, we will design several experiments on nutritional quality, postharvest, and senescence manipulation in stay-green Chinese cabbage in an attempt to elucidate these issues.

Here, a stay-green Chinese cabbage mutant was obtained by EMS mutagenesis. Genetic analysis indicated that the recessive *Brnym1* gene regulated the stay-green trait. A nonsynonymous *Brnym1* SNP was located in the stay-green domain conserved across numerous plant species. It caused a substitution from Leu to Phe. Compared with the wild type, the mutant had an extra benzene ring at its mutation site. The WT *SGR* (*BrNYM1*) of Chinese cabbage rescued the SGR-defective *nye1-1* mutant of *Arabidopsis*. Magnesium-dechelatase activity was significantly downregulated in *nym1*. Therefore, *SGR* (*BrNYM1*) influences the rate of tissue yellowing, and a defect in it results in the stay-green phenotype. The findings of the present study may be applied to improve the sensory quality and shelf life of leafy vegetables. Moreover, the stay-green gene identified herein may be invaluable in the genetic improvement and marker-assisted selection of ornamental plants and lawn grasses.

## Materials and methods

### Plant materials and mutant screening

The wild-type Chinese cabbage used here was the DH line “FT” propagated from microspore culture^38^. A stable stay-green mutant with no other pleiotropic effects was derived from EMS-mutagenized “FT” seeds and designated *nym1*. It was crossed with “FT” to produce F_1_, F_2_, and BC_1_ progeny for genetic analyses. F_2_ was also used for phenotypic characterization, mutant gene identification, and genotyping. The stay-green phenotype was screened by visual greenness assessment. Cotyledons and true leaf colors were evaluated from the seedling to adult plant stages. Stay-green plants were selected if their cotyledons or leaves remained green even until death. All plants were raised in the greenhouse of Shenyang Agricultural University, Shenyang, China, in 2018.

*Arabidopsis thaliana nye1-1* stay-green mutant seeds were provided by Benke Kuai of Fudan University, Shanghai, China. *A. thaliana* ecotype Columbia-0 (Col-0) was the control. All *A. thaliana* seeds were cold-stratified at 4 °C for 54 h before germination, sown in pots containing soil, vermiculite, and perlite at a 3:9:0.5 volumetric ratio, and incubated in a growth chamber at 22 °C under a 16 h light–8 h dark cycle and 80% RH. After germination, PCR was used to detect homozygous lines in 18-d-old *Arabidopsis nye1-1* seedlings^8^.

*Arabidopsis* seeds were surface-sterilized with 75% (v/v) ethanol for 2 min and 10% (v/v) NaClO for 8 min. After three consecutive washings with sterile water, the seeds were sown on Murashige and Skoog (MS) agar medium containing 50 mg L^−1^ hygromycin to select for transgenic plants.

### Determination of the chlorophyll content

For chlorophyll extraction, 0.1 g of the bottom leaves of adult mutant and wild-type plants (40 DAS) were cut into small pieces and placed in 10 mL of 80% (v/v) acetone in the dark until the leaves became white. Absorbances were measured at 645, 663, and 470 nm. The chlorophyll concentration was calculated according to a previously reported method^39^.

### Transmission electron microscopy (TEM) of the chloroplast

For ultrastructural analysis of the chloroplasts, the leaves of 60-d-old *nym1* and “FT” plants were cut into 1 mm × 2 mm segments, fixed with 2% (v/v) glutaraldehyde, washed with 1% (w/v) phosphate-buffered saline (PBS; 0.1 M each Na_2_HPO4∙12H_2_O and NaH_2_PO4∙2H_2_O in saline), and fixed in 1% (w/v) osmic acid (pH 7.2) for 12 h at 4 °C. The specimens were then washed with 1% (w/v) PBS; sequentially dehydrated with 50%, 70%, 80%, 90%, and 100% (v/v) acetone; embedded in epoxy resin^40^; sectioned with a microtome; and viewed under the H-7700 scanning TEM (Hitachi Ltd., Tokyo, Japan).

### Magnesium-dechelatase assay

Magnesium-dechelatase activity was evaluated with plant ELISA kits (Enzyme-Linked Biotechnology, Shanghai, China). Leaves (1 g FW) of 40-d-old *nym1* and “FT” were homogenized in 9 mL precooled PBS. The supernatants were obtained by centrifugation at 12,000 × *g* and 4 °C for 10 min and used in the assay. The protocols followed were those provided with the ELISA kit manufacturer. Calibration curves were plotted at 450 nm using a standard sample, and the sample concentrations were interpolated from them.

### Identification of the candidate gene for Brnym1

A modified MutMap method was used to identify the candidate gene for *Brnym1*^41^. DNA from 50 F_2_ plants with the stay-green phenotype was combined to make a stay-green mutant bulk (S-pool). DNA from the two parental plants and the S-pool was extracted with a DNA secure plant kit (Tiangen Biotech Ltd., Beijing, China) and resequenced with a NovaSeq 6000 sequencer (Illumina, San Diego, CA, USA). NGSQC toolkit software was used to filter the clean reads. The filtration standard was derived from a previous study^13^. The filtered clean reads from the bulk DNA pools were mapped to the *Brassica* reference genome^42^ with BWA^43^. SNPs and INDELs were detected with GATK^44^. Gene functions were annotated with ANNOVAR^45^. Variations were plotted to the genome with Circos^46^.

### SNP genotype by KASP (kompetitive allele-specific PCR) assay

KASP was used for the genotypic assay to detect cosegregation of each SNP identified by MutMap and confirm the candidate gene of *Brnym1*. Allele-specific primers bearing the FAM and HEX fluorescence probes and the common primer were designed by LGC (Laboratory of the Government Chemist, Shanghai, China) (Table S4) and used to determine the genotypes of 190 F_2_ plants. Of these, 40 were stay-green and 150 had yellow leaves. The primer mix and PCRs were programmed as recommended by LGC. The KASP thermal cycling conditions were those described in Xi et al.^47^. Fluorescence was detected with a QuantStudio 6 instrument (Applied Biosystems, Foster City, CA, USA).

### Plasmid construction and plant transformation

The full-length *BrNYM1* coding sequence was amplified using the “FT” genomic cDNA and the primers 5′-CAGTCACCTGCAAAACAACATGTGTAGTTTGTCAGCGAA-3′ and 5′-CAGTCACCTGCAAAATACACTAGAGTTTCTCCGGCTTAG-3′. The underlined sequence sections indicate *AarI* sites. The PCR products were ligated to the pGEM-T vector (Promega, Madison, WI, USA) and sequenced. The purified PCR products were digested with *AarI*. The pBWA(V)HS-ccdb vector modified from the pCAMBIA1301 vector was digested with *Eco31I*. The digested fragment was subcloned into the pBWA(V)HS binary vector driven by a cauliflower mosaic virus (CaMV) 35S promoter. The construct was sequenced and transformed into *A. tumefaciens* strain GV3101. The binary vector pBWA(V)HS:*BrNYM1* bore a hygromycin resistance gene to select transformed *Arabidopsis* lines. The floral infiltration method^48^ was used to transform *nye1-1* and Col-0 plants. The primer sequences used in this experiment are shown in Table S5.

### Phenotypic observations and verification of transgenic arabidopsis

The phenotypes of the independent T_3_ lines were observed at the seedling stage (4 weeks after sowing). The third leaves of the T_3_, Col-0, and *nye1-1* lines were incubated in darkness for 4 d to induce senescence. Transgenic lines were evaluated by qRT-PCR. The chlorophyll content was measured according to Ren et al.^8^. *AtACT* and the 35S promoter served as internal controls. Primers specific to *BrNYM1* were designed to monitor its expression in the transgenic lines.

### SGR protein sequence alignment and phylogenetic analysis

SGR protein homologs in various plants were obtained from the GenBank database (http://www.ncbi.nlm.nih.gov/BLAST) and aligned with ClustalW^49^. A phylogenetic tree was constructed with MEGA6.0 by the neighbor-joining (NJ) method based on a bootstrap test of 1000 replicates^50^.

### RNA isolation and expression analysis

Total RNA was extracted with TRIzol reagent (Invitrogen, Carlsbad, CA, USA) from young, early, and progressive naturally senescent leaves, and various organs of *nym1* and “FT” plants. First-strand cDNA synthesis and qRT-PCR were conducted as described in Wang et al.^13^. Relative expression levels were calculated by the 2^−ΔΔCt^ method. *ACT* (actin) was used as the internal control. Gene-specific primers were designed according to the *nym1* and “FT” cDNA sequences. The primer sequences used in this experiment are shown in Table S5.

### Subcellular localization of BrNYM1 proteins

ProCAMV35S:BrNYM1:GFP (GFP) constructs were built to determine the subcellular localizations of the BrNYM1 proteins. The coding regions of the *BrNYM1* sequences were amplified without stop codons using the primers BrNYM1GFP-F-XbaI and BrNYM14GFP-R-SalI. They were cloned into the *C*-terminal GFP fusion vector pBI221 driven by the 35S promoter. DNA sequencing verified the constructs. Vector-bearing, 35S-driven GFP was the negative control. Subcellular localization of the BrNYM1 proteins was performed according to Yoo et al.^51^. Transient GFP expression was detected in *A. thaliana* mesophyll cell protoplasts under a confocal laser-scanning microscope (Leica Microsystems, Wetzlar, Germany). GFP fluorescence signals were visualized at an excitation wavelength of 488 nm. The emission signals were detected at 643–730 nm for chlorophyll autofluorescence and at 495–530 nm for GFP. The primer sequences used in this experiment are shown in Table S5.

## Supplementary information

supplementary information
