# Supplementary material for: Defect in Brnym1, a magnesium-dechelatase protein, causes a stay-green phenotype in an EMS-mutagenized Chinese cabbage (Brassica campestris L. ssp. pekinensis) line
Source: Hortic Res. 2020 Jan 1;7:8. doi: 10.1038/s41438-019-0223-6 (PMC6944686; doi:10.1038/s41438-019-0223-6)
Supplement: Supplementary file 1 — supplementary information [file 41438_2019_223_MOESM1_ESM.docx]

**Defect in Brnym1, a magnesium-dechelatase protein, causes a stay-green phenotype in a Chinese cabbage (*Brassica campestris* L. ssp. *pekinensis*) EMS mutagenesis line**

Nan Wang, Yun Zhang, Shengnan Huang, Zhiyong Liu, Chengyu Li, Hui Feng **^*^**

*Department of Horticulture, Shenyang Agricultural University, Shenyang, China*

**Supplementary information**

This file contains Figure S1-S3 and Supplementary Tables S1 – S5.

**Fig.S1** Characterization of siliques at harvesting time in WT (‘FT’) and *nym1* mutant plants


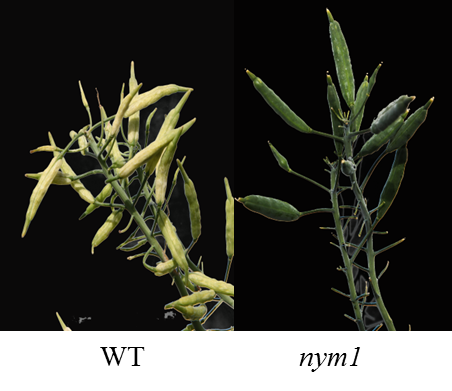


**Fig. S2** Verification of the candidate gene by F_2_ population using KASP technology

: CC, : CT, : TT


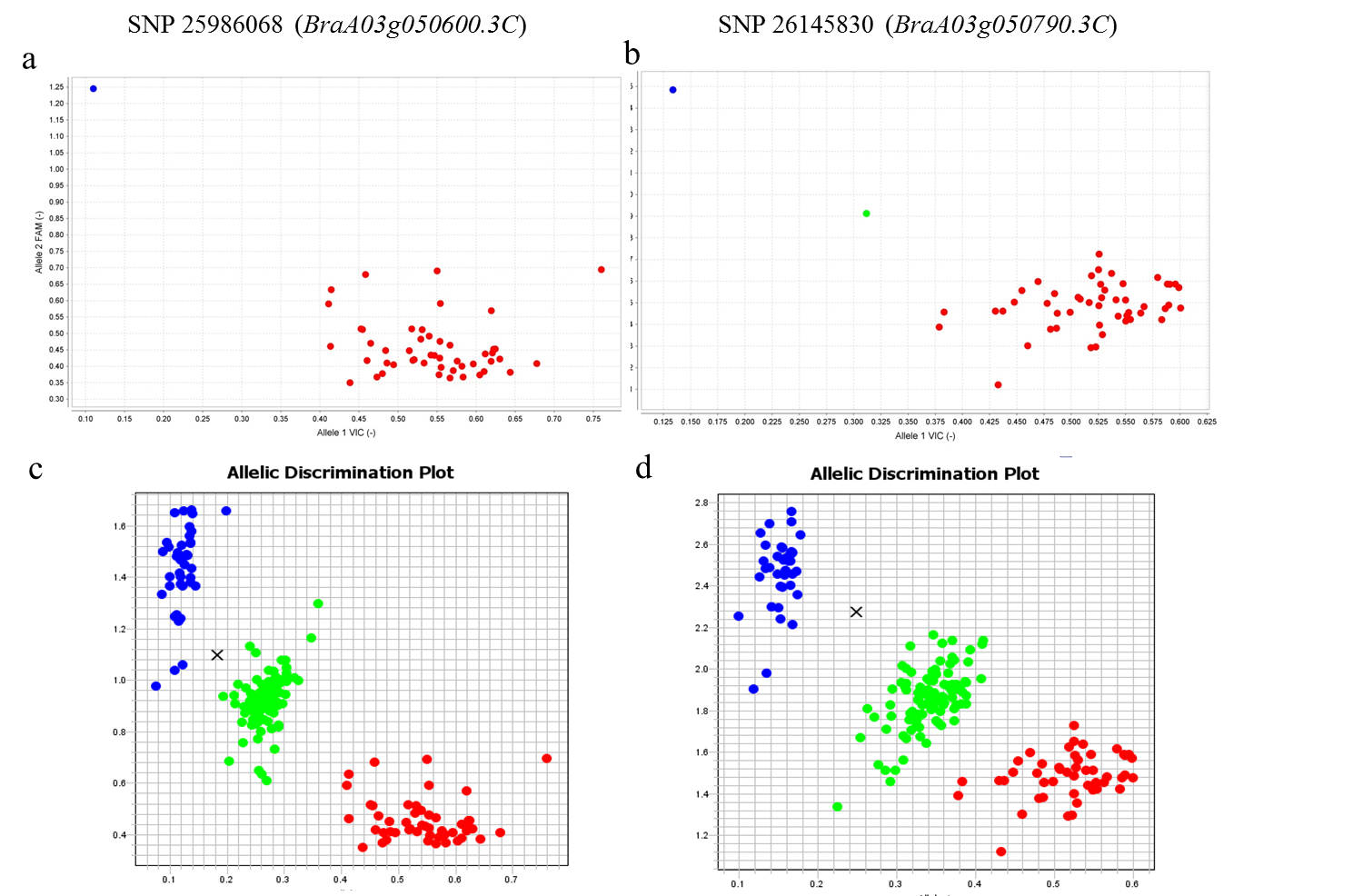


**Fig. S3** Sequence alignment of AtSGR and BrSGR


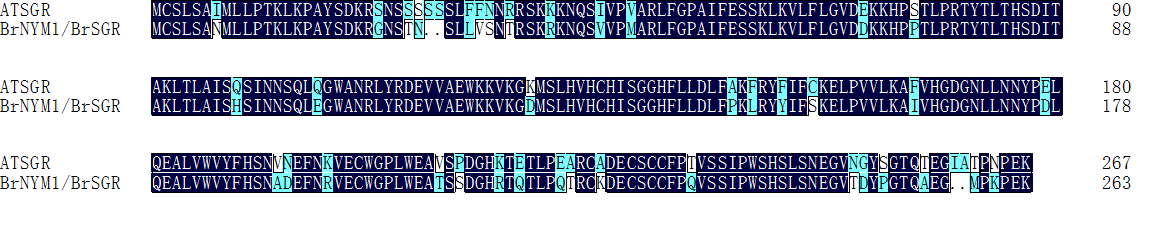


**Table S1** Genetic analyses of the stay-green character in the progeny of parental ‘FT’ and *nym1*

| Generations | Total | Yellow | Stay-green | Segregation Ratio | χ2 |
| --- | --- | --- | --- | --- | --- |
| P_1_(‘FT’) | 50 | 50 | 0 |  |  |
| P_2_(*nym1*) | 50 | 0 | 50 |  |  |
| F_1_(P_1_×P_2_) | 98 | 98 | 0 |  |  |
| F_1_(P_2_×P_1_) | 96 | 96 | 0 |  |  |
| BC_1_(F_1_×‘FT’) | 75 | 75 | 0 |  |  |
| BC_1_(F_1_×*nym1*) | 109 | 57 | 52 | 1.09:1 | 0.23 |
| F_2_ | 762 | 576 | 186 | 3.09:1 | 0.14 |

**Table S2** List of candidate SNPs

| Chr | Pos | Ref | WT | Mut | SNP  index | Location | Gene ID | Exon ID | Nucleotide change | Amino acid change | Mutation type |
| --- | --- | --- | --- | --- | --- | --- | --- | --- | --- | --- | --- |
| A03 | 25665854 | G | G | A | 1 | intergenic | BraA03g050000.3C(dist=10384),BraA03g050010.3C(dist=2900) | -- | -- | -- | -- |
| A03 | 25727778 | G | G | A | 1 | upstream;downstream | BraA03g050120.3C(dist=480);BraA03g050130.3C(dist=933) | -- | -- | -- | -- |
| A03 | 25855135 | G | G | A | 1 | upstream | BraA03g050320.3C(dist=336) | -- | -- | -- | -- |
| A03 | 25952225 | G | G | A | 1 | downstream | BraA03g050500.3C(dist=760) | -- | -- | -- | -- |
| A03 | 25986068 | G | G | A | 1 | exonic | BraA03g050600.3C | exon3 | c.C385T | p.L129F | nonsynonymous SNV |
| A03 | 26011192 | G | G | A | 1 | intergenic | BraA03g050620.3C(dist=14858),BraA03g050630.3C(dist=4304) | -- | -- | -- | -- |
| A03 | 26145830 | G | G | A | 1 | exonic | BraA03g050790.3C | exon1 | c.G415A | p.E139K | nonsynonymous SNV |
| A03 | 26227418 | G | G | A | 1 | intronic | BraA03g050950.3C | -- | -- | -- | -- |
| A03 | 26371372 | G | G | A | 0.9821429 | intergenic | BraA03g051180.3C(dist=9893),BraA03g051190.3C(dist=2964) | -- | -- | -- | -- |
| A03 | 26377063 | G | G | A | 0.9487179 | intergenic | BraA03g051200.3C(dist=1699),BraA03g051210.3C(dist=5111) | -- | -- | -- | -- |

**Table S3** Genotyping results of SNP25986068 and SNP26145830

| **F_2_ population** | **Phenotype** | **Genotype-SNP 25986068** | **Genotype-SNP 26145830** | **F_2_ population** | **Phenotype** | **Genotype-SNP 25986068** | **Genotype-SNP 26145830** |
| --- | --- | --- | --- | --- | --- | --- | --- |
| 1 | stay-green | T:T | T:T | 97 | leaf yellow | T:C | T:C |
| 2 | stay-green | T:T | T:T | 98 | leaf yellow | C:C | C:C |
| 3 | stay-green | T:T | T:T | 99 | leaf yellow | T:C | T:C |
| 4 | stay-green | T:T | T:T | 100 | leaf yellow | T:C | T:C |
| 5 | stay-green | T:T | T:T | 101 | leaf yellow | T:C | T:C |
| 6 | stay-green | T:T | T:T | 102 | leaf yellow | T:C | T:C |
| 7 | stay-green | T:T | T:T | 103 | leaf yellow | T:C | T:C |
| 8 | stay-green | T:T | T:T | 104 | leaf yellow | C:C | C:C |
| 9 | stay-green | T:T | C:T | 105 | leaf yellow | T:C | T:C |
| 10 | stay-green | T:T | T:T | 106 | leaf yellow | T:C | T:C |
| 11 | stay-green | T:T | T:T | 107 | leaf yellow | T:C | T:C |
| 12 | stay-green | T:T | T:T | 108 | leaf yellow | T:C | T:C |
| 13 | stay-green | T:T | T:T | 109 | leaf yellow | C:C | C:C |
| 14 | stay-green | T:T | T:T | 110 | leaf yellow | T:C | T:C |
| 15 | stay-green | T:T | T:T | 111 | leaf yellow | T:C | T:C |
| 16 | stay-green | T:T | T:T | 112 | leaf yellow | T:C | T:C |
| 17 | stay-green | T:T | T:T | 113 | leaf yellow | T:C | T:C |
| 18 | stay-green | T:T | T:T | 114 | leaf yellow | C:C | C:C |
| 19 | stay-green | T:T | T:T | 115 | leaf yellow | T:C | T:C |
| 20 | stay-green | T:T | T:T | 116 | leaf yellow | C:C | C:C |
| 21 | stay-green | T:T | T:T | 117 | leaf yellow | T:C | T:C |
| 22 | stay-green | T:T | T:T | 118 | leaf yellow | T:C | T:C |
| 23 | stay-green | T:T | T:T | 119 | leaf yellow | C:C | C:C |
| 24 | stay-green | T:T | T:T | 120 | leaf yellow | T:C | T:C |
| 25 | stay-green | T:T | T:T | 121 | leaf yellow | C:C | C:C |
| 26 | stay-green | T:T | T:T | 122 | leaf yellow | T:C | T:C |
| 27 | stay-green | T:T | T:T | 123 | leaf yellow | C:C | C:C |
| 28 | stay-green | T:T | T:T | 124 | leaf yellow | T:C | T:C |
| 29 | stay-green | T:T | T:T | 125 | leaf yellow | T:C | T:C |
| 30 | stay-green | T:T | T:T | 126 | leaf yellow | C:C | C:C |
| 31 | stay-green | T:T | T:T | 127 | leaf yellow | T:C | T:C |
| 32 | stay-green | T:T | T:T | 128 | leaf yellow | C:C | C:C |
| 33 | stay-green | T:T | T:T | 129 | leaf yellow | T:C | T:C |
| 34 | stay-green | T:T | T:T | 130 | leaf yellow | C:C | C:C |
| 35 | stay-green | T:T | T:T | 131 | leaf yellow | T:C | T:C |
| 36 | stay-green | T:T | T:T | 132 | leaf yellow | T:C | T:C |
| 37 | stay-green | T:T | T:T | 133 | leaf yellow | T:C | T:C |
| 38 | stay-green | T:T | T:T | 134 | leaf yellow | C:C | C:C |
| 39 | stay-green | T:T | T:T | 135 | leaf yellow | T:C | T:C |
| 40 | stay-green | T:T | T:T | 136 | leaf yellow | T:C | T:C |
| 41 | stay-green | T:T | T:T | 137 | leaf yellow | C:C | C:C |
| 42 | stay-green | T:T | T:T | 138 | leaf yellow | T:C | T:C |
| 43 | stay-green | T:T | T:T | 139 | leaf yellow | C:C | C:C |
| 44 | stay-green | T:T | T:T | 140 | leaf yellow | T:C | T:C |
| 45 | stay-green | T:T | T:T | 141 | leaf yellow | T:C | T:C |
| 46 | stay-green | T:T | T:T | 142 | leaf yellow | C:C | C:C |
| 47 | stay-green | T:T | T:T | 143 | leaf yellow | T:C | T:C |
| 48 | stay-green | T:T | T:T | 144 | leaf yellow | C:C | C:C |
| 49 | stay-green | T:T | T:T | 145 | leaf yellow | T:C | T:C |
| 50 | leaf yellow | C:C | C:C | 146 | leaf yellow | T:C | T:C |
| 51 | leaf yellow | C:C | C:C | 147 | leaf yellow | T:C | T:C |
| 52 | leaf yellow | C:C | C:C | 148 | leaf yellow | T:C | T:C |
| 53 | leaf yellow | T:C | T:C | 149 | leaf yellow | T:C | T:C |
| 54 | leaf yellow | T:C | T:C | 150 | leaf yellow | C:C | C:C |
| 55 | leaf yellow | T:C | T:C | 151 | leaf yellow | T:C | T:C |
| 56 | leaf yellow | C:C | C:C | 152 | leaf yellow | C:C | C:C |
| 57 | leaf yellow | T:C | T:C | 153 | leaf yellow | T:C | T:C |
| 58 | leaf yellow | T:C | T:C | 154 | leaf yellow | T:C | T:C |
| 59 | leaf yellow | C:C | C:C | 155 | leaf yellow | T:C | T:C |
| 60 | leaf yellow | T:C | T:C | 156 | leaf yellow | T:C | T:C |
| 61 | leaf yellow | T:C | T:C | 157 | leaf yellow | T:C | T:C |
| 62 | leaf yellow | C:C | C:C | 158 | leaf yellow | T:C | T:C |
| 63 | leaf yellow | T:C | T:C | 159 | leaf yellow | C:C | C:C |
| 64 | leaf yellow | T:C | T:C | 160 | leaf yellow | T:C | T:C |
| 65 | leaf yellow | C:C | C:C | 161 | leaf yellow | T:C | T:C |
| 66 | leaf yellow | T:C | T:C | 162 | leaf yellow | T:C | T:C |
| 67 | leaf yellow | T:C | T:C | 163 | leaf yellow | T:C | T:C |
| 68 | leaf yellow | T:C | T:C | 164 | leaf yellow | T:C | T:C |
| 69 | leaf yellow | T:C | T:C | 165 | leaf yellow | T:C | T:C |
| 70 | leaf yellow | C:C | C:C | 166 | leaf yellow | T:C | T:C |
| 71 | leaf yellow | C:C | C:C | 167 | leaf yellow | C:C | C:C |
| 72 | leaf yellow | C:C | C:C | 168 | leaf yellow | T:C | T:C |
| 73 | leaf yellow | T:C | T:C | 169 | leaf yellow | T:C | T:C |
| 74 | leaf yellow | T:C | T:C | 170 | leaf yellow | T:C | T:C |
| 75 | leaf yellow | T:C | T:C | 171 | leaf yellow | T:C | T:C |
| 76 | leaf yellow | C:C | C:C | 172 | leaf yellow | T:C | T:C |
| 77 | leaf yellow | T:C | T:C | 173 | leaf yellow | C:C | C:C |
| 78 | leaf yellow | T:C | T:C | 174 | leaf yellow | T:C | T:C |
| 79 | leaf yellow | C:C | C:C | 175 | leaf yellow | T:C | T:C |
| 80 | leaf yellow | T:C | T:C | 176 | leaf yellow | T:C | T:C |
| 81 | leaf yellow | C:C | C:C | 177 | leaf yellow | T:C | T:C |
| 82 | leaf yellow | T:C | T:C | 178 | leaf yellow | T:C | T:C |
| 83 | leaf yellow | T:C | T:C | 179 | leaf yellow | C:C | C:C |
| 84 | leaf yellow | T:C | T:C | 180 | leaf yellow | C:C | C:C |
| 85 | leaf yellow | T:C | T:C | 181 | leaf yellow | T:C | T:C |
| 86 | leaf yellow | T:C | T:C | 182 | leaf yellow | T:C | T:C |
| 87 | leaf yellow | T:C | T:C | 183 | leaf yellow | T:C | T:C |
| 88 | leaf yellow | T:C | T:C | 184 | leaf yellow | C:C | C:C |
| 89 | leaf yellow | T:C | T:C | 185 | leaf yellow | T:C | T:C |
| 90 | leaf yellow | T:C | T:C | 186 | leaf yellow | T:C | T:C |
| 91 | leaf yellow | T:C | T:C | 187 | leaf yellow | C:C | C:C |
| 92 | leaf yellow | T:C | T:C | 188 | leaf yellow | T:C | T:C |
| 93 | leaf yellow | T:C | T:C | 189 | leaf yellow | T:C | T:C |
| 94 | leaf yellow | T:C | T:C | 190 | leaf yellow | T:C | T:C |
| 95 | leaf yellow | T:C | T:C | 191 | leaf yellow | T:C | T:C |
| 96 | leaf yellow | T:C | T:C | 192 | leaf yellow | T:C | T:C |

**Table S4** List of primer sequence of KASPs

| **ID** | **Primer_AlleleFAM** | **Primer_AlleleHEX** | **Primer_Common** | **AlleleFAM** | **AlleleHEX** |
| --- | --- | --- | --- | --- | --- |
| *BraA03g050600_3C* | AATGTGGCAGTGGACGTGAAG | CTAATGTGGCAGTGGACGTGAAA | AAGTGGTAGCAGAATGGAAGAAAGTGAAA | C | T |
| *BraA03g050790_3C* | GAAGGTGACCAAGTTCATGCTAGTGACGCAACAAGTCAAGAGG | GAAGGTCGGAGTCAACGGATTAGTGACGCAACAAGTCAAGAGA | CTGCATCATTGTTCTTCTTTGGA | C | T |

**Table S5** Sequences of the primers used in this study

| Primer name | Primer Sequences (5′–3′) | Length of PCR products(bp) | Tm (°C) |
| --- | --- | --- | --- |
| BrNYE1GFP-F-XbaI | GCTCTAGAATGTGTAGTTTGTCAGCGAA | 792 |  |
| BrNYE14GFP-R-SalI | ACGCGTCGACGAGTTTCTCCGGCTTAG |  | 58 |
| BrACT-F | CGAAACAACTTACAACTCCA |  |  |
| BrACT-R | CTCTTTGCTCATACGGTCA | 150 | 58 |
| qSGR-F | GCATCCACCAACGCTCC |  |  |
| qSGR-R | GCCTATTTGCCCATCCTTC | 175 | 58 |
| AtACT-F  AtACT-R | ATTACCCGATGGGCAAGTCA  CAGCGATACCTGAGAACATAGTGG | 150 | 58 |
